# Effect of the Different Dietary Supplements on the Average Surface Roughness and Color Stability of Direct Restorative Materials Used in Pediatric Dentistry

**DOI:** 10.3390/children11060645

**Published:** 2024-05-27

**Authors:** Nagehan Aktaş, Yasemin Akın, Cenkhan Bal, Mehmet Bani, Merve Bankoğlu Güngör

**Affiliations:** 1 Department of Pediatric Dentistry, Faculty of Dentistry, Gazi University, Ankara 06560, Türkiye; yasemin.akdemir@gazi.edu.tr (Y.A.); mehmetbani@gazi.edu.tr (M.B.); 2 Department of Pediatric Dentistry, Gülhane Dentistry Faculty, Health Sciences University, Ankara 06018, Türkiye; cenkhanbal@gmail.com; 3 Department of Prosthetic Dentistry, Faculty of Dentistry, Gazi University, Ankara 06560, Türkiye; mervegungor@gazi.edu.tr

**Keywords:** dietary supplements, discoloration, pediatric dentistry, restorative materials

## Abstract

Increased surface roughness and discoloration of the direct restorative materials used in pediatric patients affect the longevity of restorations and impair children’s oral health. Many factors can alter these properties. One of these factors is the intake of dietary supplements. It is crucial to predict the properties of restorative materials when exposed to dietary supplements to maintain the dental care of children. Thus, this study aimed to investigate the effect of various syrup-formed dietary supplements on the average surface roughness and color stability of current restorative materials used in pediatric dentistry. Seven different restorative materials (conventional glass ionomer [Fuji IX GP], resin-modified glass ionomer, [Fuji II LC], zirconia-reinforced glass ionomer [Zirconomer Improved], polyacid-modified composite resin [Dyract^®^XTRA], bulk-fill glass hybrid restorative [Equia Forte HT Fill], conventional resin composite [Charisma Smart], and resin composite with reactive glass fillers [Cention N]) were tested. The specimens prepared from each type of restorative material were divided into five subgroups according to dietary supplements (Sambucol Kids, Resverol, Imunol, Umca, and Microfer). These specimens were immersed daily in supplement solution over a period of 28 days. Surface roughness and color difference measurements were performed at baseline and at the 7th and 28th days. The color difference and Ra values showed that there was an interaction among the type of restorative material, type of dietary supplement, and immersion time factors (*p* < 0.05). Whereas lower Ra values were found in the composite resin group, the highest Ra values were found in the conventional glass ionomer group. All supplements caused increasing color difference values, and Resverol and Umca showed higher discoloration values above the clinically acceptable threshold. The intake of dietary supplement type, the immersion time of the dietary supplement, and the restorative material type affected the surface roughness and color stability of the tested direct restorative materials. All of the experimental groups showed higher Ra values than clinically acceptable surface roughness values (0.2 µm). The color difference values also increased with the immersion time.

## 1. Introduction

In pediatric dentistry, the composition of restorative materials is important in maintaining children’s oral health in the long-term, and also the properties of the restorative material affect the esthetic appearance. Composite resins, glass ionomer cements (GICs), and compomers are commonly utilized restorative materials to treat children to achieve satisfactory results [1,2]. Glass ionomer restorations are the most popular direct restorations for pediatric patients, particularly in non-load-bearing areas. These cements have two components, such as fluoroaluminosilicate glass and poly acrylic acid, a chemical bonding mechanism to harden tooth structures, a similar thermal expansion coefficient to dentin, and easy manipulation procedures [3]. Although these have favorable properties, low wear resistance, and fracture strength, their long setting time, rough surface, and sensitivity to moisture limit their clinical applications [4,5]. GICs are often improved to address their drawbacks by adding some reinforcing particles into the material, such as silver alloy particles, resin monomers, glass fillers, and zirconia ceramics [6]. Resin-modified GICs (RMGICs) are restorative materials with resin components and were developed to overcome the problems of conventional glass ionomers, such as moisture sensitivity and low initial mechanical strength [7]. Zirconia-added GICs are known as Zirconomer (white amalgam) [4], in which the glass component undergoes precise micronization to achieve ideal particle size and properties, and the uniform incorporation of zirconia fillers enhances the mechanical properties [8,9,10]. Another GIC-based material is Equia Forte, which is a viscous form of GICs. The encapsulation of the material avoids the need for manual mixing, the addition of highly reactive smaller particles to the powder strengthens the material, and covering the restoration with a newly developed nanofilled coating material improves its properties [3,5,11]. The newly introduced restorative material Cention N is an alkasite material that releases acid-neutralizing ions incorporated into the resin matrix [12,13]. As acid attacks occur, the restorative material’s alkaline filler releases hydroxide ions, regulating the pH [13,14]. Manufacturers assert that this ormocer formulation offers superior mechanical and physical characteristics, such as esthetics, adhesion, and fluoride release [12]. It combines the properties of both amalgam and GIC [14]. Besides these restoratives, resin composites are often used in dentistry because of their ability to mimic the shape and color of natural teeth. Their physical and chemical properties allow the composite restoration to endure daily oral challenges and preserve its integrity, luster, and color [15].

Dietary supplements are defined as products that are taken orally and contain a “dietary ingredient” by the United States Food and Drug Administration (FDA). These supplements are typically used to maintain or improve overall health and meet nutritional needs. They contain constituents that enhance the diet, such as vitamins, minerals, amino acids, botanicals, and herbs. Supplements come in many forms, including tablets, soft gels, and liquids [16,17]. The severity of an infection is determined by immune competence. When various substances, for instance, vitamins and minerals, are inadequate, virus infections may cause immune system damage. Dietary supplements like those described above might increase the immune response [18]. It has been reported that many natural dietary supplements have an effect against various types of respiratory viruses, and therefore these supplements can be used as an adjuvant therapy together with antiviral drugs, including in the treatment of the coronavirus disease of 2019 (COVID-19) [19]. Furthermore, children need sufficient amounts of vitamins and minerals to maintain healthy functions, growth, and development. Since the body cannot produce enough of these nutrients necessary for the body in some cases, they must be taken from external supplements when necessary [20]. The pandemic has reignited interest in dietary supplements worldwide, and sales of supplements that claim to boost immune health have increased [17]. In a study investigating the relationship between mothers’ fears of COVID-19 and their children’s attitudes toward using food supplements, it was reported that almost 60% of children took dietary supplements during this period [21].

The ingredients used in drug formulations in the form of syrup/liquid include sugars, acids, buffering agents, and coloring agents in the form of oil and/or water-soluble agents. In addition, the long-term use of these drugs may cause adverse effects such as erosion, internal/external staining on tooth surfaces, and surface degradation of dental restorations due to their endogenous pH and high titratable acidity [1,20,22,23]. The surface roughness and color stability in restorative materials used in pediatric dentistry are essential to increase the longevity of the restoration and to maintain children’s oral health. A higher surface roughness of the restoration materials is associated with increased plaque accumulation, adhesion of caries-causing bacteria, and periodontal inflammation, and the critical surface roughness value for bacterial adhesion is 0.2 µm [24]. Discoloration of the restoration materials may occur due to exposure to staining foods and beverages, liquids, or medications. The replacement of the discolored restoration in children increases treatment costs, costs parents more time, and may cause compliance problems with pediatric patients [25,26]. Previous studies have focused mostly on the effect of pediatric drugs in the form of syrup/liquid (antitussive, analgesic, antibiotic, antihistaminic, and multivitamin) on the discoloration effects of restorative materials [1,2,11,20,25,26,27,28,29,30,31,32,33]. Frequent dental visits for restoration replacements can lead to issues such as increased costs and potential increases in behavioral management/dental anxiety in young patients [26].

Although increased use of these supplements has been reported, to the best of our knowledge, no studies are currently focusing on the effect of the use of dietary supplements on the average surface roughness and color stability of direct restorative materials used in pediatric dentistry. Therefore, the aim of this study was to evaluate the effect of various liquid dietary supplements on the average surface roughness and color stability of current restorative materials used in pediatric dentistry. For this aim, seven different direct restoratives (conventional glass ionomer, resin-modified glass ionomer, zirconia-reinforced glass ionomer, polyacid-modified composite resin, bulk-fill glass hybrid restorative, conventional resin composite, and resin composite with reactive glass fillers) commonly used in pediatric patients were selected, and five syrup-formed dietary supplements with different compositions (Sambucol, Resverol, Imunol, Umca, and Microfer) were tested in the present study. The null hypothesis was that there would be no differences among the different materials after the application of the dietary supplements in terms of average surface roughness and color stability.

## 2. Materials and Methods

To investigate the effect of restorative material type and dietary supplement type for three different immersion times on the surface roughness values and for two different immersion times on the color difference values, the minimum required sample sizes were calculated with the G-Power program ver. 3.1.9.4. Assuming a medium effect size (f = 0.25), a Type I error rate of 0.05, a power of 0.95, and a correlation value of 0.30 between the repeated measures were used, and the minimum total sample size required was determined to be 210 with 6 specimens per group (35 groups) for surface roughness, and the minimum total sample size required was determined to be 245 with 7 specimens per group (35 groups) for color difference.

### 2.1. Specimen Preparation

In the present study, the effects of five different liquid dietary supplements on the average surface roughness and color stability of seven different restorative materials were investigated. The properties of the tested restorative materials and the dietary supplements are presented in Table 1 and Table 2.

A total of 350 specimens (*n* = 50 for each restorative material) were prepared according to the manufacturer’s instructions. Round metal molds (15 mm in diameter and 2 mm in thickness) were used to prepare the specimens. The metal molds were placed on a mylar strip (Universal strip, EDD Dental, Hofheim, Germany) on a glass slab to achieve a uniformly flat surface [2]. The restorative materials were filled into the molds and covered with mylar strips to prevent the formation of an oxygen inhibition layer, and then the resin-based materials were polymerized with a light-emitting diode (LED) curing device (Valo, Ultradent, South Jordan, UT, USA) for 20 s. The specimen preparation and polymerization procedures recommended by the manufacturer for each restorative material are presented in Table 3. Both surfaces of the specimens were polished with aluminum-oxide-impregnated disks (Sof-Lex XT, 3M ESPE, St. Paul, MN, USA). Then, the specimens were cleaned in distilled water by using an ultrasonic device. The specimens were numbered and kept in distilled water in the incubator at 37 °C for 24 h to complete the polymerization [2,11,33].

### 2.2. Immersion Time

**Figure 1:**
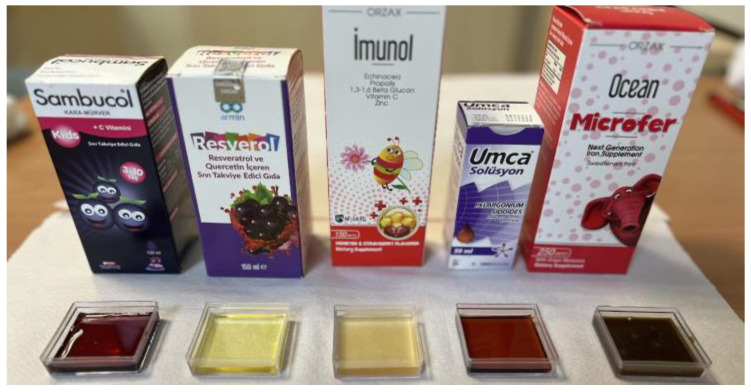
Immersion medium used in the study.

In this study, the specimens from each type of restorative material were categorized into five subgroups (*n* = 10) based on the dietary supplement (Sambucol kids, Resverol, Imunol, Umca, and Microfer) (Figure 1). The pH levels of the dietary supplements were measured by using a pH meter (Orion 5-Star Plus pH/ORP/ISE/Conductivity/DO Benchtop Meter, Thermo Scientific Corp., Waltham, MA, USA). The employed immersion cycling protocol was designed to mirror the real-life intake patterns of the dietary supplements [20]. These specimens were immersed daily for 2 min in 10 mL of the dietary supplement solution, maintaining a period of 24 h between each immersion over a span of 28 days [20]. Post-immersion, the specimens were rinsed and stored in deionized water until the following cycle. The syrups for immersion were replaced for each cycle. The specimens were maintained in deionized water throughout the experimental period (28 days), with daily renewal of the water. The color measurements and average surface roughness were evaluated at each disc-shaped specimen’s baseline and at the 7th and 28th days.

### 2.3. Measurement of Color Parameters

The specimens were rinsed with distilled water for 5 s and air-dried. The color parameters (L, a, and b) of each specimen were measured (C_0_) under a standard illuminant D65 (MASTER TL-D Super 80 18 W/865 1SL; Philips, Eindhoven, Holland) with a spectrophotometer (Konica Minolta; Minolta Konica, Tokyo, Japan). The measuring characteristics of the spectrophotometer were standard illuminant D65, illumination geometry d/8, 10 colorimetric standard observer, SCE mode, and measurement area of 8 mm in diameter. All measurements were performed in the same color measurement box covered with a neutral gray background.

The L (lightness, from 0 = black to 100 = white), a (from a =green to +a = red), and b (from b = blue to +b = yellow) values of the specimens were obtained as a value composed of three axes [11]. For measuring the color parameters, the device was calibrated with its own calibration plate before each experimental group. A total of thirty measurements were performed for each experimental group. Mean values were provided for each specimen. The measurements were repeated after the 7th (C_7_) and 28th (C_28_) days, and the color differences (ΔE_ab_) between C_0_–C_7_ and C_0_–C_28_ were calculated with the following equation [2,11,26,34,35]:

∆E_ab_ = [(∆L)^2^ + (∆a)^2^ + (∆b)^2^] ^½^

The color difference values of the experimental groups were compared with the perceptibility (1.2) and acceptability (2.7) thresholds [36,37,38]. The perceptibility threshold is the minimal color variation that an observer can perceive and refers to a situation in which 50% of observers notice a difference in color between two objects. The acceptability threshold is the acceptable color difference perceived by 50% of observers. The difference between these two thresholds indicates the allowable deviation from the perceptible difference that still results in an acceptable color match [36,37,38].

### 2.4. Measurements of Average Surface Roughness (Ra)

The average surface roughness (Ra) of all specimens was measured with a contact stylus profilometer (Marsurf M 300 C, MarSurf RD 18 C, stylus PHT 6–350/2 μm, Mahr GmbH; Göttingen, Germany) at a speed of 0.5 mm/s and with a movement distance of 1.75 mm. The device was calibrated with its calibration plate (PRN-10; Mahr GmbH; Göttingen, Germany) before measuring each group. Measurements were performed 3 times from the surface of each specimen in 3 different directions. The mean Ra value was calculated for each specimen. The surface roughness values were compared with the clinically acceptable surface roughness value (0.2 µm) [24,39].

### 2.5. Statistical Analyses

All statistical analyses were performed with the SPSS statistical software program (IBM SPSS Statistics for Windows, v20.0; IBM Corp., Armonk, NY, USA). The distributions of the average surface roughness and color difference data were tested with the Shapiro–Wilk test and analyzed with three-way repeated measures of analysis of variance (ANOVA). The factors were the type of restorative material, type of dietary supplement, and immersion time. The Greenhouse–Geisser correction was used when the sphericity assumption was violated. Pairwise comparisons were tested with Tukey’s HSD tests. The results were considered as significant for *p* < 0.05.

## 3. Results

The pH levels of the tested dietary supplements were ordered as follows: 2.96 (Resverol), 3.07 (Imunol), 3.20 (Sambucol kids), 3.87 (Microfer), and Umca (4.10), from highest to lowest.

**Figure 2:**
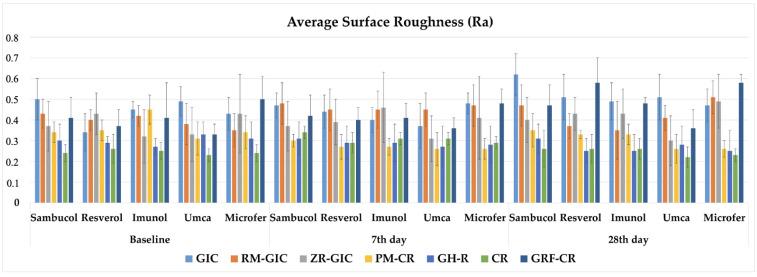
The graphical view of the Ra values of the experimental groups.

The three-way repeated measures of ANOVA results of the Ra values showed that there was an interaction among the type of restorative material, type of dietary supplement, and immersion time factors (*p* < 0.05). The mean values, standard deviations (±), and comparisons of the Ra data of the experimental groups are shown in Table 4. The graphical view of the Ra values of the experimental groups are shown in Figure 2. The Ra values of all experimental groups were above the clinically acceptable surface roughness value (0.2 µm). It was observed that surface roughness values were increased with the immersion time. Lower Ra values were found in the CR group among the experimental groups. 

**Figure 3:**
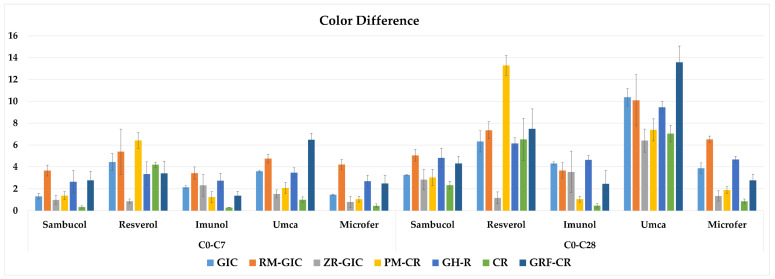
The graphical view of the color difference values of the experimental groups.

The three-way repeated measures of ANOVA results of the color difference values showed that there was an interaction among the type of restorative material, type of dietary supplement, and immersion time factors (*p* < 0.05). The mean values, standard deviations (±), and comparisons of the color difference data of the experimental groups are shown in Table 5. The graphical view of the color difference values of the experimental groups is shown in Figure 3. The color difference values after 7 days of immersion were above the clinically perceptible threshold value (1.2) in all the dietary supplement groups of GIC, RM-GIC, GH-R, and GRF-CR. However, the PM-CR and CR groups in Imunol, the ZR-GIC group in Resverol, and the CR group in Microfer showed clinically imperceptible color difference values after 28 days of immersion. The results showed that Umca caused the highest discoloration values in most of the groups except for the Resverol group of PM-CR after the 28th day of immersion.

## 4. Discussion

In the present study, the effect of the different dietary supplements on the average surface roughness and the color difference values of different direct restoratives used for pediatric patients was investigated. The results demonstrated that the type of restorative material, the dietary supplement, and the immersion time were effective on the surface roughness and the color difference values of the tested materials; thus, the null hypothesis was rejected.

Various restorative materials with different microstructures have been introduced to pediatric dentistry due to the increasing demands for esthetic and minimally invasive dental treatments. Therefore, clinicians should consider the esthetics and mechanical durability of the restoration when selecting the appropriate material for the treatment of children and should balance and control the factors affecting the oral health of the children in the long-term. In the present study, these newly introduced materials (Zirconomer Improved, Equia Forte HT Fill, Cention N) were included in the experimental groups, and their results were compared with conventional restorative materials (Fuji IX GP, Fuji II LC, Dyract^®^XTRA, Charisma Smart) used in pediatric dentistry.

One of the factors affecting the clinical success and longevity of direct restorations is the intake of colored and acidic dietary supplements. These supplements particularly came to the forefront with the COVID-19 pandemic, and it was observed in the literature research that the effects of these supplements on newly developed restorative materials used in pediatric dentistry had not been extensively assessed. Many liquid medications for children have a high sugar content and acidity. These characteristics of medications have raised questions in several studies [40,41,42] about the potential risk of dental caries or erosion. Moreover, the use of such medications can also cause discoloration on both teeth and restorations and alter their surface properties [33]. Seven different restorative materials with different microstructures and polymerizing methods (conventional glass ionomer, resin-modified glass ionomer, zirconia-reinforced glass ionomer, polyacid-modified composite resin, bulk-fill glass hybrid restorative, conventional resin composite, and resin composite with reactive glass fillers) and five syrup-formed dietary supplements with different contents (Sambucol, Resverol, Imunol, Umca, and Microfer) were tested in the present study. The effects of these supplements on the selected restorative materials were investigated in terms of surface roughness and color difference. Although these supplements are generally used for only a short period of time, their repetitive use can have a significant effect on the properties of both enamel and restorative materials [34]. In the present study, the supplements were evaluated for 28 days; however, some of the supplements may be used for a longer or shorter period of time. It was decided by the researchers of the present study that 28 days should represent an approximate time for the tested dietary supplements. No regular brushing of the specimens was performed. After each immersion cycle, the specimens were stored in distilled water; thus, it was aimed to mimic drinking water or rinsing the mouth after the intake of the dietary supplement. The specimens were subjected to brushing at baseline and after the 7th and 28th days before the measurements.

The use of dietary supplements in a syrup form with low pH levels can be erosive for dental hard tissues; furthermore, supplements can potentially soften restorative materials, leading to decreased wear resistance, increased surface roughness, and discoloration [20,34]. The pH values of the dietary supplements selected for this study were not included by the manufacturers. The reported pH value for the Sambucol syrup was 4.02, which was below the critical pH value of 5.5 [20]. To determine the pH levels of the tested dietary supplements in the present study, the pH levels of the supplements were measured by using a pH meter. The results demonstrated that all the dietary supplements had pH levels lower than the critical pH value (5.5).

A higher surface roughness of restorative materials and tooth structures is associated with plaque accumulation. Because microorganisms can easily colonize on rough surfaces, periodontal diseases and caries can be observed in dental tissues adjacent to these surfaces [43,44,45]. Nalwade et al. [23] investigated the effects of pediatric liquid medications on the surface roughness of Zirconomer, composite resin, and GIC. It was stated that the surface roughness value was significantly lower with Zirconomer compared to composite material and GIC. However, in another study, Sharafeddin et al. [4] reported that GIC, RMGIC, and Zirconomer had 1.46 ± 0.18, 0.49 ± 0.11, and 1.73 ± 0.21 µm surface roughness values, respectively. It was reported that Zirconomer had the highest Ra value compared with the other cements, and the higher surface roughness value of Zirconomer could arise from its larger particle size [46]. Particle size is an important factor for the surface roughness of GICs [4], and RMGIC has a smaller particle size than Zirconomer and GIC [46,47]. Furthermore, the resin content of RMGIC decreases its surface roughness [48]. In the present study, the Ra values were increased with the immersion time in most of the groups, and the values differed according to the restorative material and dietary supplement type. GIC showed a higher Ra value, and CR showed a lower Ra value. However, the clinically acceptable surface roughness value was reported as 0.2 µm [39], and all of the experimental groups showed higher Ra values than this value in the present study. Thus, mechanical polishing of these restorations in clinical follow-ups and applying a coating, if recommended, may be beneficial to reduce surface roughness.

Perceptible differences in color of restorative materials are associated with the esthetics of restorations. Discoloration can lead to parental concern and negatively impact social interactions among preschool children [35]. Thus, replacement of discolored restorations may become necessary in some clinical cases. The color stability of restorative material is explained by the color differences between two periods of time [36]. This value is determined with the ΔE_ab_ and ΔE_00_ values, which are calculated by using the CIELab and CIEDE2000 formulas. The ΔE_ab_ values, which represent the distance between the color coordinates (L*, a*, and b*) [35], are used in most studies in pediatric dentistry [1,2,11,26,29,34,49,50]. Thus, to compare the results with the relevant studies, the mean ΔE_ab_ values of the experimental groups were determined in the present study. The color difference results were also compared with the perceptibility (1.2) and acceptability (2.7) thresholds [24,36].

Tüzüner et al. [26] evaluated the effects of different pediatric drugs on the difference in color of polyacid-modified composite resin, composite resin, and glass ionomer cement. The results showed that only the restorative material was effective on the color difference values. The composite resin showed the highest discoloration among the tested materials. It was reported that GICs showed acceptable color stability compared to composite or compomer materials. In contrast, both the restorative material and the dietary supplement type were effective on the color difference values in the present study. These different results may have arisen from the difference in the formulations of the tested medications. The color of the dietary supplement may be another factor in the discoloration. Sambucol and Umca are claret red/purple, Resverol and Imunol are light yellow, and Microfer is brown. Higher color difference values were observed for the GRF-CR group of Umca (13.6) and the PM-CR group of Resverol (13.3) after 28 days of immersion. However, this result suggests that the composition of the dietary supplement is more influential than its color on the restoration’s color, depending on the restorative type. In another study, Çınar et al. [34] reported that acidic dietary supplements caused color differences in resin composites, and they also impaired the staining resistance of the material. However, the effect differed due to the type of restorative material and dietary supplement. In the present study, the pH values of the tested dietary supplements were also found to be below the critical pH value (5.5). All the supplements caused increasing color difference values with the immersion time; moreover, Resverol and Umca showed higher discoloration values above the clinically acceptable threshold. Discoloration may also occur due to restorative-material-dependent factors, such as the properties, degree of conversion, and hydrophilicity of the resin matrix, the size and quantity of the filler particles, the mode and depth of polymerization, and the surface properties of the material [51,52,53,54]. Materials with a lower filler content and rougher surfaces are more susceptible to discoloration [55]. Larger filler particle sizes can lead to increased water absorption through the polymer chains, which can also contribute to discoloration [52]. This is because water sorption impairs the interface between the resin matrix and filler particles, and it causes microcracks, interfacial gaps, stain penetration, and discoloration [56]. Faghihi et al. [11] investigated the effect of analgesics, antibiotics, anticonvulsants, and multivitamins on the difference in color of conventional reinforced GIC, resin-reinforced GIC, flowable composite resin, and composite resin. The GIC had higher color difference values compared to the composite resin in all the immersion conditions. Accordingly, the GIC groups showed higher color difference values than the CR groups in all the dietary supplements except for Resverol, in which the results were similar for the GIC and CR groups. Resverol had the lowest pH value among the tested supplements. Therefore, it is thought that the acidic concentration of this supplement had a greater degradation effect on the materials.

GICs are more prone to discoloration due to several factors, including the porosity and water absorption of the glass particles, dehydration after setting and drying, and the presence of microcracks that can lead to color differences [57]. Tian et al. [58] studied the microstructure and Hertzian indentation failure in biocompatible glass ionomer cements and reported that cracks grew to link pores while propagating along glass–matrix interfaces. Furthermore, resin composites are hydrophobic materials, and they exhibit superior color stability and stain resistance compared to hydrophilic materials like GICs and compomers [59].

Faghihi et al. [11] reported that a higher color difference value was observed in RMGIC compared to GICs and composite resins. In the present study, RMGIC showed higher color difference values than GIC in most of the dietary supplement groups. The polymerization process can be effective in observing different results. Although RMGICs and GICs contain a similar ion-releasing mechanism, RMGICs have smaller filler particles. The initial setting reaction of RMGICs is light-cured, which starts the polymerization of the resin, and an acid–base reaction subsequently occurs in the presence of water, helping to complete the setting process and enhancing the material’s properties [60]. In the present study, the GH-R group (bulk-fill glass hybrid restorative system; Equia Forte Fill) showed higher color difference values than the CR and PMCR (polyacid-modified composite resin; Dyract) groups. Yıldırım and Uslu [33] tested the effects of five different pediatric drugs (antibiotic, analgesic, common cold syrup, cough syrup, and an iron-and-vitamin formula) and tooth brushing on the color stability of three restorative materials (polyacid-modified composite resin, compomer; glass hybrid; and glass carbomer). The highest color difference value was found in the polyacid-modified composite resin/non-brushing group immersed in the iron-and-vitamin formula. Thus, regular brushing after the intake of dietary supplements may be beneficial in reducing the discoloration of restorative materials. In the present study, the color difference values of the RMGIC were higher than the GH-R groups except for the group with immersion in Imunol. Faghihi et al. [11] also stated that bulk-fill glass hybrid restoratives had lower color difference values than RMGICs. The reduced tendency to discoloration of this new material may be due to the nanofilled coating material. This coating material enhances the initial stabilization of the filling material during polymerization and promotes increased infiltration and closure of superficial defects in GIC [11].

The present study has limitations. In-vitro experimental conditions might not fully replicate real oral conditions, where restorative materials are regularly exposed to colorants from food and drinks and saliva containing enzymes, proteins, and ions that could impact their color stability. Additional research is necessary to evaluate the surface irregularities, polymerization levels, water absorption, finishing, and polishing of restorative materials.

## 5. Conclusions

The intake of dietary supplements (supplement type), the immersion time of the dietary supplement, and the restorative material type affected the surface roughness and color stability of the tested direct restorative materials.

The surface roughness values were generally increased with the immersion time. GIC showed a higher Ra value, and CR showed a lower Ra value. However, all of the experimental groups showed higher Ra values than clinically acceptable surface roughness value (0.2 µm).

The color difference values were also increased with the immersion time. All the supplements caused increasing color difference values, and Resverol and Umca showed higher discoloration values above the clinically acceptable threshold (2.7).

## Figures and Tables

**Table 1:** Restorative materials used in the study.

Material	Classification	Composition	Manufacturer
Fuji IX GP	Conventional glass ionomer cement	Powder: aluminum–calcium–lanthanum fluorosilicate glass, acrylic acid, maleic acid; Liquid: poly(alkenoic acid) tartaric acid, water	3M ESPE, St. Paul, MN, USA
Fuji II LC	Resin-modified glass ionomer cement	Powder: fluoroaluminosilicate glass, HEMA, urethane dimethacrylate, water, photoinitiator (camphorquinone); Liquid: poly(acrylic acid)	GC, Tokyo, Japan
Zirconomer Improved	Zirconia-reinforced glass ionomer cement	Zirconium oxide (nano-sized zirconia filler particles ranging from 96.5% to 98.5%), glass powder, tartaric acid, polyacrylic acid, and deionized water	Shofu Inc., Ashford, UK
Dyract^®^XTRA	Polyacid-modified composite resin (compomer)	UDMA, TCB resin, TEGDMA, trimethacrylate resin, camphorquinone, ethyl-4-dimethylaminobenzoate, BHT, UV stabilizer, strontium-alumino-sodiumfluorophosphor-silicate glass, highly dispersed silicon dioxide, strontium fluoride, iron oxide and titanium dioxide pigments (mean filler size: 0.8, filler volume 47%)	Dentsply, Konstanz, Germany
Equia Forte HT Fill	Bulk-fill glass hybrid restorative system	Powder: fluoroaluminosilicate glass, polyacrylic acid, iron oxide; Liquid: polybasic carboxylic acid, water	GC, Tokyo, Japan
Charisma Smart	Conventional composite resin	Bis-EMA, HEDMA, TEGDMA, barium aluminium fluoride glass (0.02–2 μm), pyrogenic silicon dioxide (0.02–0.07 μm)	Kulzer, Hanau, Germany
Cention N	“Alkasite” (composite resin with reactive glass fillers)	Powder: barium aluminum silicate glass, ytterbium trifluoride, isofiller, calcium barium aluminum fluorosilicate glass, calcium fluoro silicate glass; Liquid: urethane dimethacrylate, tricyclodecandimethanol dimethacrylate, tetramethyl-xylylene, diurethane dimethacrylate, polyethylene glycol 400, dimethacrylate, ivocerin, hydroperoxide	Ivoclar Vivadent, Schaan, Lichtenstein

**Table 2:** Dietary supplements used in the study.

Product	Composition	Manufacturer
Sambucol kids	Glucose syrup, black elderberry juice concentrate, L-ascorbic acid (vitamin C). Acidity regulator: citric acid. Preservative: potassium sorbate	PharmaCare, Karlsruhe, Germany
Resverol	Resveratrol, quercetin, vitamin C	Armin, İzmir, Türkiye
Imunol	Echinacea extract, 1.3–1.6 beta glucan, zinc, propolis, vitamin C	Orzax, İstanbul, Türkiye
Umca	Pelargonium sidoide liquid extract, maltodextrin, xylitol, glycerol, citric acid anhydrous, potassium sorbate, xanthan gum, pure water	ISO Arzneimittel GmbH&Co., Karlsruhe, Germany
Microfer	Iron (lipophere ‘microencapsulated iron source’)	Orzax, İstanbul, Türkiye

**Table 3:** Specimen preparation and polymerization procedures recommended by the manufacturer for the restorative materials tested in this study.

Material	Specimen Preparation	Polymerization Procedures
Fuji IX GP	A standard powder/liquid ratio of 1 level scoop of powder and 1 drop of liquid (3.6 g/1.0 g) was placed on the pad. The powder was divided into two parts with the help of a plastic spatula. The first portion was mixed with all the liquid for 10 s. The remaining powder was added, and the whole sample was mixed for 15–20 s.	Self-curing material, setting time of 2 min 20 s.
Fuji II LC	The capsule was shaken to loosen the powder inside the capsule, and its piston was pushed until it was aligned with the main body to activate the capsule. The capsule was placed in an amalgamator and mixed for 10 s. The mixed capsule was loaded into the GC capsule applicator, and the application was made.	Light-curing for 20 s using a visible light-curing device (LED/Halogen > 700 mW/cm^2^).
Zirconomer Improved	Two parts powder and one drop (standard powder/liquid ratio: 3.6 g/1.0 g) of liquid were dispersed onto the mixing pad. The dispensed powder was divided into 2 equal portions; the first half was added to the distributed liquid and mixed with a plastic spatula for 5–10 s. Then, the remaining half was added and mixed for a total of 30 s until it reached a thick paste consistency.	Self-curing material, setting time of 3 min (from the end of mixing)
Dyract^®^XTRA	The compule tip was inserted into the notched opening of the gun. The material was placed by applying constant pressure to the gun.	Curing to 10 s (depth of at least 2 mm) using a lamp with an output over 500 mW/cm^2^
Equia Forte HT Fill	The capsule was shaken to loosen the powder. Then, the plunger was pressed and held firmly for 2 s. It was mixed in the amalgamator for 10 s. The capsule was immediately inserted into the applicator and applied within 10 s.	Setting time of 2 min 30 s (from the start of mixing)
Charisma Smart	The ready-made material in the syringe was applied directly.	Curing time of 20 sn (for maximum layer thickness 2 mm) for blue-light-curing units (wavelength peak at 450–480 nm; light output of 1550–600 mW/cm^2^
Cention N	One measuring spoon of powder and one drop of liquid were used as the mixing ratio. The powder and liquid were taken onto a mixing pad, and the powder was divided into two equal-sized pieces with a plastic spatula. The first part was mixed first, and then the remaining powder was added. It was mixed again for 45–60 s until a homogeneous consistency was obtained.	* Self-curing material, with light-curing option Setting time of 5 min (from the start of mixing) The restoration can be optionally light-cured after placement (for ≥500 mW/cm^2^ light intensity, exposure time 40 sn; for >1000 mW/cm^2^ light intensity, exposure times 20 sn).

* Self-curing option was selected.

**Table 4:** Mean values and standard deviations (±) of the Ra data of the experimental groups.

Material	Baseline					7th Day					28th Day				
	Sambucol	Resverol	Imunol	Umca	Microfer	Sambucol	Resverol	Imunol	Umca	Microfer	Sambucol	Resverol	Imunol	Umca	Microfer
GIC	0.5 (±0.10) A a 2	0.34 (±0.09) BC b 3	0.45 (±0.04) A a 12	0.49 (±0.07) A a 1	0.43 (±0.08) A a 1	0.47 (±0.06) A ab 2	0.44 (±0.08) A abc 2	0.40 (±0.06) A bc 2	0.37 (±0.11) B c 2	0.48 (±0.05) A a 1	0.62 (±0.10) A a 1	0.51 (±0.11) A b 1	0.49 (±0.09) A b 1	0.51 (±0.11) A b 1	0.47 (±0.08) A b 1
RM-GIC	0.43 (±0.07) AB a 1	0.40 (±0.05) AB ab 12	0.42 (±0.05) A a 1	0.38 (±0.10) B ab 2	0.35 (±0.08) B b 2	0.48 (±0.10) A a 1	0.45 (±0.10) A a 1	0.45 (±0.09) A a 1	0.45 (±0.08) A a 1	0.47 (±0.10) A a 1	0.47 (±0.10) B ab 1	0.37 (±0.06) BC c 2	0.35 (±0.14) BC c 2	0.41 (±0.06) B bc 12	0.51 (±0.08) AB a 1
ZR-GIC	0.37 (±0.12) BC ab 1	0.43 (±0.10) A a 1	0.32 (±0.13) B b 2	0.33 (±0.13) B b 1	0.43 (±0.19) A a 12	0.37 (±0.12) BC ab 1	0.39 (±0.11) A bc 1	0.46 (±0.17) A c 1	0.31 (±0.11) BC a 1	0.41 (±0.20) A bc 2	0.40 (±0.11) BC b 1	0.43 (±0.08) B bc 1	0.43 (±0.12) AB bc 1	0.30 (±0.12) CD a 1	0.49 (±0.13) A c 1
PM-CR	0.34 (±0.05) C b 1	0.35 (±0.05) ABC b 1	0.45 (±0.07) A a 1	0.31 (±0.08) B b 1	0.34 (±0.08) B b 1	0.30 (±0.03) C a 1	0.27 (±0.06) B a 2	0.27 (±0.04) B a 2	0.26 (±0.08) C a 1	0.26 (±0.05) B a 2	0.35 (±0.08) CD ac 1	0.33 (±0.02) CD b 12	0.33 (±0.05) CD ab 2	0.26 (±0.07) DE a 1	0.26 (±0.04) C bc 2
GH-R	0.30 (±0.08) CD a 1	0.29 (±0.03) CD a 1	0.27 (±0.04) B a 1	0.33 (±0.06) B a 1	0.31 (±0.08) BC a 1	0.31 (±0.08) C a 1	0.29 (±0.08) B a 1	0.29 (±0.09) B a 1	0.27 (±0.10) C a 1	0.28 (±0.09) B a 1	0.31 (±0.07) DE a 1	0.25 (±0.06) E a 1	0.25 (±0.08) E a 1	0.28 (±0.09) DE a 1	0.25 (±0.10) C a 1
CR	0.24 (±0.04) D a 2	0.26 (±0.07) D a 1	0.25 (±0.04) B a 2	0.23 (±0.03) C a 2	0.24 (±0.04) C a 1	0.34 (±0.03) C a 1	0.29 (±0.05) B a 1	0.31 (±0.03) B a 1	0.31 (±0.03) BC a 1	0.29 (±0.03) B a 1	0.26 (±0.09) E a 2	0.26 (±0.07) DE a 1	0.26 (±0.05) DE a 12	0.22 (±0.05) E a 2	0.23 (±0.03) C a 1
GRF-CR	0.41 (±0.10) B c 1	0.37 (±0.08) AB bc 2	0.41 (±0.17) A c 1	0.33 (±0.05) B b 1	0.50 (±0.11) A a 2	0.42 (±0.10) AB bc 1	0.40 (±0.06) A b 2	0.41 (±0.07) A abc 1	0.36 (±0.05) B b 1	0.48 (±0.07) A c 2	0.47 (±0.10) B b 1	0.58 (±0.12) A a 1	0.48 (±0.03) A b 1	0.36 (±0.09) BC c 1	0.58 (±0.04) B a 1

Same uppercase letters indicate that the Ra values of the restorative material groups were not significantly different in the same dietary supplement and immersion time groups (*p* > 0.05). Same lowercase letters indicate that the Ra values of the dietary supplement groups were not significantly different in the same restorative material and immersion time groups (*p* > 0.05). Same numbers indicate that the Ra values of the immersion time groups were not significantly different in the same restorative material and dietary supplement groups (*p* > 0.05).

**Table 5:** Mean values and standard deviations (±) of the color difference data of the experimental groups.

Material	C_0_–C_7_					C_0_–C_28_				
	Sambucol	Resverol	Imunol	Umca	Microfer	Sambucol	Resverol	Imunol	Umca	Microfer
GIC	1.32 (±0.25) C d 2	4.45 (±0.76) C b 2	2.15 (±0.14) B c 2	3.61 (±0.08) C a 2	1.46 (±0.05) C d 2	3.27 (±0.04) B d 1	6.33 (±0.96) C b 1	4.32 (±0.14) AB c 1	10.37 (±0.80) C a 1	3.88 (±0.48) B cd 1
RM-GIC	3.67 (±0.49) A cd 2	5.39 (±2.06) B a 2	3.44 (±0.55) A d 1	4.75 (±0.39) B b 2	4.21 (±0.45) A bc 2	5.06 (±0.55) A d 1	7.35 (±0.78) B b 1	3.68 (±0.70) B c 1	10.09 (±2.34) BC a 1	6.53 (±0.28) A b 1
ZR-GIC	0.98 (±0.42) C bc 2	0.88 (±0.19) E c 1	2.32 (±0.99) BC a 2	1.53 (±0.37) DE b 2	0.80 (±0.49) DE c 1	2.85 (±0.94) BC b 1	1.17 (±0.55) D c 1	3.55 (±1.88) B b 1	6.41 (±1.05) E a 1	1.35 (±0.47) CD c 1
PM-CR	1.38 (±0.37) C c 2	6.42 (±0.72) A b 2	1.24 (±0.51) D c 1	2.08 (±0.49) D a 2	1.05 (±0.26) CD c 2	3.05 (±0.74) BC c 1	13.29 (±0.91) A a 1	1.06 (±0.26) C e 1	7.39 (±1.01) D b 1	1.89 (±0.30) C d 1
GH-R	2.65 (±1.02) B b 2	3.36 (±1.09) D a 2	2.74 (±0.69) C b 2	3.49 (±0.43) C a 2	2.70 (±0.55) B b 2	4.83 (±0.88) A c 1	6.15 (±0.50) C b 1	4.65 (±0.39) A c 1	9.45 (±0.55) B a 1	4.68 (±0.27) B c 1
CR	0.34 (±0.11) D c 2	4.2 (±0.24) C a 2	0.28 (±0.02) E c 1	1.01 (±0.24) E b 2	0.44 (±0.18) E bc 1	2.35 (±0.35) C c 1	6.52 (±1.94) C a 1	0.46 (±0.21) C b 1	7.05 (±0.75) DE a 1	0.86 (±0.21) D b 1
GRF-CR	2.79 (±0.79) B d 2	3.43 (±1.08) D b 2	1.37 (±0.4) D c 2	6.48 (±0.6) A a 2	2.5 (±0.74) B d 1	4.31 (±0.64) A d 1	7.49 (±1.81) B b 1	2.46 (±1.21) D c 1	13.57 (±1.48) A a 1	2.79 (±0.54) E c 1

Same uppercase letters indicate that the color difference values of the restorative material groups were not significantly different in the same dietary supplement and immersion time groups (*p* > 0.05). Same lowercase letters indicate that the color difference values of the dietary supplement groups were not significantly different in the same restorative material and immersion time groups (*p* > 0.05). Same numbers indicate that the color difference values of the immersion time groups were not significantly different in the same restorative material and dietary supplement groups (*p* > 0.05).

## Data Availability

The datasets used and analyzed during the present study are available from the corresponding author upon reasonable request. The datasets are not readily available because technical limitation.
